# Inter-Individual Variability in Fear of Humans and Relative Brain Size of the Species Are Related to Contemporary Urban Invasion in Birds

**DOI:** 10.1371/journal.pone.0018859

**Published:** 2011-04-19

**Authors:** Martina Carrete, José L. Tella

**Affiliations:** 1 Department of Conservation Biology, Estación Biológica de Doñana, Consejo Superior de Investigaciones Científicas, Sevilla, Spain; 2 Department of Physical, Chemical and Natural Systems, University Pablo de Olavide, Sevilla, Spain; University of Utah, United States of America

## Abstract

**Background:**

Urbanization is the most prevailing cause of habitat transformation worldwide, differing from others by its intense levels of human activity. Despite its obvious impact on wildlife, it is still unclear why and how some species are able to adapt to urban settings. One possibility is that fear of humans and vehicles could preclude most species from invading cities. Species entering urban environments might be those that are more tolerant of human disturbance (i.e., *tame species*). Alternatively or in addition, urban invaders could be a fraction of *variable species*, with “tame” individuals invading urban habitats and other individuals remaining in rural areas.

**Methodology:**

Using the contemporary urban invasion by birds in a recently established South American city, we tested both hypotheses by relating interspecific differences in invasiveness to their flight initiation distances (i.e., the distances at which birds flee from approaching cars, FID), as well as to their relative brain size (RBS), a correlate of measures of behavioral flexibility.

**Principal Findings:**

Urban invasiveness was not significantly related to species' average rural FIDs but positively related to their RBS and inter-individual variability in FID. Moreover, FIDs were consistently lower in urban than in rural conspecifics, and the FIDs of urban individuals were within the lower-range distribution of their rural conspecifics. RBS indirectly influenced urban invasion through its positive effect on inter-individual variability in FID.

**Conclusions/Significance:**

Urban invaders do not appear to be individuals from apparently *tame* species, but rather *tame* individuals from species with a variable response regarding fear of people. Given the positive relationship between RBS and inter-individual variability in FID, our results suggest that behavioural flexibility should be regarded as a specific trait encompassing variability among individuals. Further research is needed to ascertain the neurophysiological mechanisms underlying the relationship between brain size and inter-individual variability in behavioural traits.

## Introduction

Urbanization can be considered one of the most severe and lasting forms of land-use modification which is occurring unchecked worldwide [1]. Approximately half of the human population currently lives in cities, with the proportion of those residing in urban environments increasing rapidly [2]; thus, an intensification of the current biodiversity crisis associated with this urban expansion into native ecosystems is expected [1]. Therefore, the urbanization process is a challenge for biodiversity conservation [3] but it also presents a unique scenario for evolutionary biologists to study specific traits that make some species better at colonizing new niches than others [4]. Birds offer a good study model for this purpose because while many species are negatively affected by the current spread of urbanization [5]–[6] others such as the house sparrow (*Passer domesticus*) are almost exclusively urban dwellers.

Despite its intuitive significance, fear of humans has been largely overlooked as a behavioural trait precluding the entrance of some species into urban environments [7]–[8]. Humans are potential predators of birds, to the point that their flight initiation distances (i.e. the distance at which birds flee from approaching humans, hereafter FID) have been considered as measures of antipredatory responses [9] and of anthropogenic stressors [10]. In fact, there is ample evidence that pedestrian activity causes disturbance, measured as FIDs, in natural habitats [11]. Besides people, cars and other vehicles are omnipresent in urban areas, and can seriously disturb neophobic species and/or individuals. In this context, the notions of neophobia and neophilia (i.e., the spontaneous aversion or attraction of an animal to a food item, object, or place because it is novel [12]) are known to be important since they may play a decisive role in the ability of an individual to face new situations and may greatly influence an animal's apparent cognitive ability [12]. Car traffic is known to affect breeding densities and activity patterns of birds [13], also causing direct mortality through road kills. A recent review on the effects of road traffic on the distribution and abundance of animals shows that species are negatively affected due to direct disturbance or car casualties [14]. In cities, cars usually travel at low velocity and it could be expected that disturbance effects could be more important than direct mortalities.

Here, we tested two non-alternative hypotheses to explain avian urban invasions related to fear of humans and their accompanying vehicles. First, birds entering urban environments might belong to *tame species*, i.e. those more tolerant of human disturbance [7]–[8]. Individuals trade-off early flight for other activities such as resource acquisition, reproduction or rest, so bird species showing lower FIDs would be more able to cope with human disturbance and invade cities than species that do not [7]. Under this hypothesis, we predicted that if urban invaders belong to the group of *tame species* then the main factor explaining variability in invasiveness among species should be their mean FID (M_FID_) measured in rural (i.e. ‘natural’) habitats. A within-species corollary prediction is that FIDs of urban individuals would not significantly differ from FIDs of conspecifics living in rural habitats. Second, individuals entering urban areas could belong to *variable species*, i.e. those species whose individuals respond differently to human presence. In this case, urban invasion would be mainly possible by tame individuals from species showing larger inter-individual variability in their response to human disturbance, measured as the coefficient of variation of FID (CV_FID_) in rural habitats. As a within-species corollary prediction, urban individuals should show shorter FIDs than their rural conspecifics. This idea derives from the disturbance-induced habitat selection hypothesis recently proposed by Carrete & Tella [15] and from a recent study testing the importance of individual variability in FID in urban invasiveness in the Old World [8]. Carrete & Tella [15] showed a strong individual consistency in FIDs (repeatability: 0.84–0.92) of burrowing owls (*Athene cunicularia*), suggesting that individuals may distribute themselves among breeding sites depending on their susceptibility to human disturbance. This has been recently supported by Evans *et al.* [16], who found differences in behavioural syndromes linked to FID between rural and urban song sparrows (*Melospiza melodia*). Moreover, Møller [8] showed a significant contribution of variability in FIDs in explaining urban invasiveness in the Old World. Although this result is of great importance in the understanding of urban invasiveness, the current set of urban species might have resulted from multiple processes of colonization, adaptation and extinction likely undergone by urban bird populations in European countries, where the thousand-year-old cities may have experienced changes in human attitudes towards birds as well as in habitat conditions. Thus, as stated long ago by Diamond [4], the study of urban invasions should also be carried out in areas where urbanization processes are recent and thus contemporary evolution is actually at work.

Perhaps the colonization by bird species of these newly urbanized areas is better explained by some yet unexplored components of behavioural flexibility [17]–[18] rather than by their fear of humans [8], so we also considered this possibility. Different evidence suggests that large brains, relative to body size, can confer advantages to individuals to modify their behaviour in potentially adaptive ways [18]–[19]. Such enhanced behavioural flexibility is predicted to lend fitness benefits to individuals facing novel or altered environmental conditions, an idea known as the brain size-environmental change hypothesis [20]. Larger brains allow animals to process, integrate, and store more information about their environment, enhancing the capacity of individuals to modify or acquire new behaviours (innovations) in flexible ways [19]–[23]. In this sense, species with relatively large brains would tend to be more successful in establishing themselves in new environments by enhancing their innovation propensity [17]. Under this behavioural flexibility hypothesis, our prediction is that species with relatively larger brains will be better at invading urban sites than species with smaller brain sizes.

To properly assess the relative importance of fear of humans and relative brain size (RBS) in urban invasiveness, we simultaneously tested the contribution of mean FID, within-species FID variability and RBS through Generalized Linear Mixed Models. We also included potentially confounding variables previously shown to be related to avian invasiveness such as dietary and habitat generalism [17], [24], environmental tolerance [25], and abundance of the species in rural habitats [17], [24], as well as body size because of its positive relationship to FID [9], [26]. To avoid biases derived from the long-term coexistence between people and birds in ancient cities, we focused on a recently urbanized system to test behavioural traits underlying urban invasion as a contemporary process [4].

## Results

**Figure 1:**
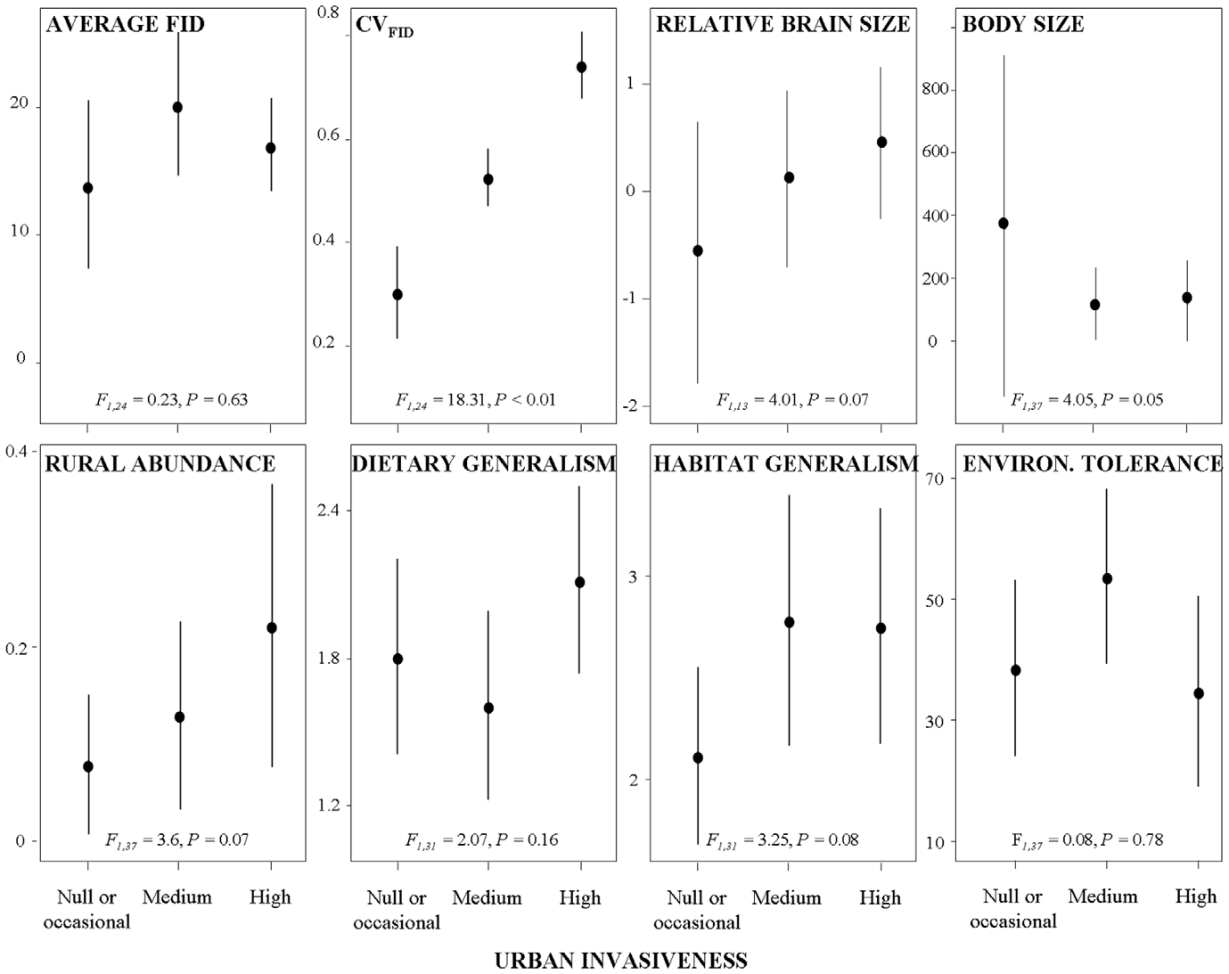
Mean (±95% CI) of average and variability in flight initiation distances (CV_FID_) measured in rural areas, relative brain size, body size, rural abundance, dietary and habitat generalism, and environmental tolerance of 42 species related to their different urban invasiveness. Statistical results are controlled for Family and Order fitted as nested random terms in models.

Urban invasiveness of the study species was scored into three rough categories (null or occasional, medium and high) based on their within-species relative abundances in rural and urban areas. Univariate analyses showed that urban invasiveness was positively related to the relative brain size (RBS, calculated as the residuals of a log-log linear regression of brain mass against body mass) and the inter-individual variability in flight initiation distance (CV_FID_, measured as the coefficient of variation in FID) of 42 species measured in rural habitats, but not to their average FID (M_FID_, Fig. 1). Invasiveness was also marginally related to their between-species relative abundance in surrounding rural areas and body size, the most common and smaller species being more likely to invade cities. Species invading urban habitats were also those showing higher habitat generalism (obtained as the number of the major habitat types recorded in the literature for each species), a relationship that was marginally significant. Dietary generalism (obtained as the number of the major food types recorded in the literature for each species) and environmental tolerance (calculated as the whole latitudinal distribution of each species) did not show clear relationships to urban invasiveness (Fig. 1).

**Figure 2:**
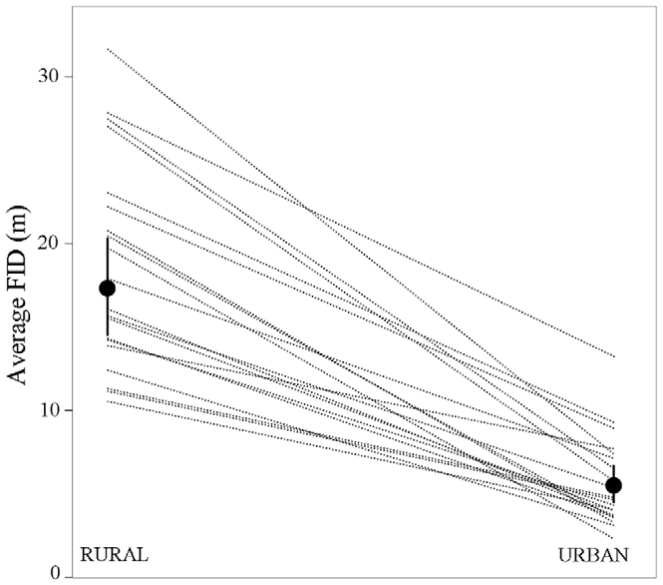
Intra-specific comparison of flight initiation distances (mean ±95% CI) for 20 species measured both in rural and urban habitats. Each line connects one species.

**Figure 3:**
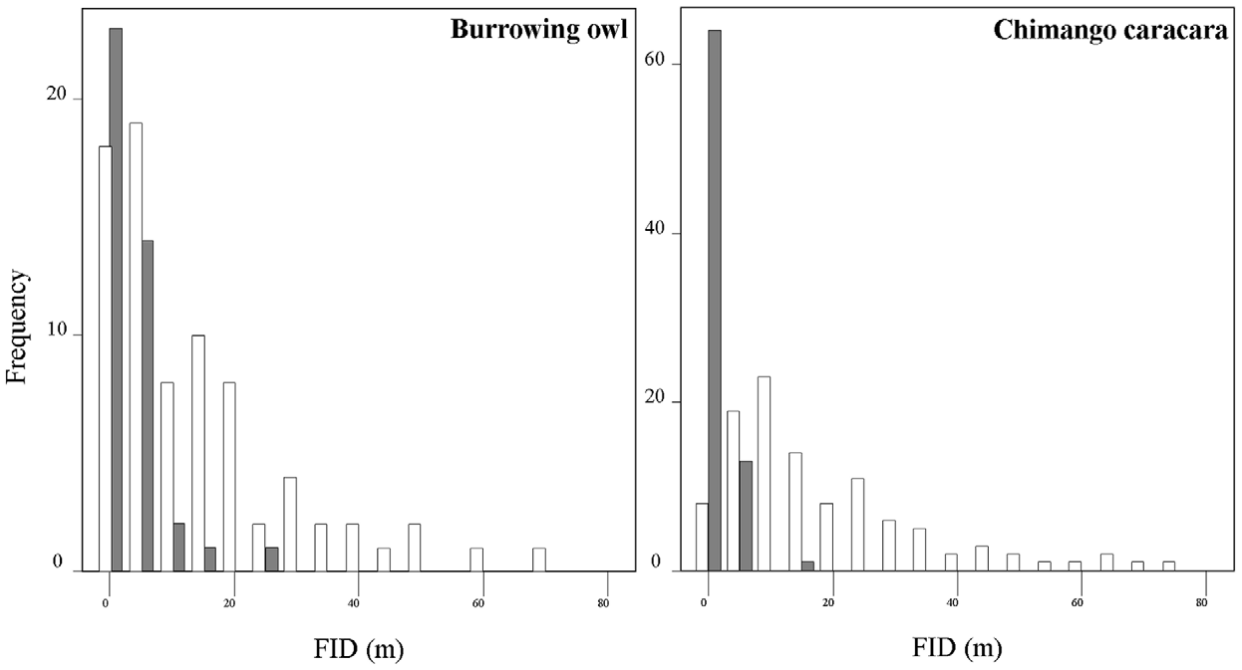
Distribution of flight initiation distances of urban (black bars) and rural (white bars) individuals, as exemplified by the burrowing owl *Athene cunicularia* (n = 119) and the chimango caracara *Milvago chimango* (n = 185).

Among urban invading species, an intraspecific comparison of 20 species present in urban and rural habitats consistently showed lower FIDs in urban than in rural conspecifics in all examined species (Paired t-test, Z = −4.02, P<0.0001; Fig. 2). Moreover, the distributions of FIDs of urban individuals were all within the lower-tail range distributions of their conspecifics living in rural habitats (see the two species with larger sample sizes in Fig. 3), suggesting that tame individuals belonging to species consisting of a gradient between tame and less tame individuals, are those entering into urban areas. Notably, one of the species shown in Fig. 3 is the burrowing owl, from which we are confident we sampled different, territorial birds and in which we previously demonstrated high within-individual repeatability in FID (see Methods). All these results offer support to our predictions of higher urban invasiveness among species showing variable FIDs and larger brains, but not among apparently tame species.

**Figure 4:**
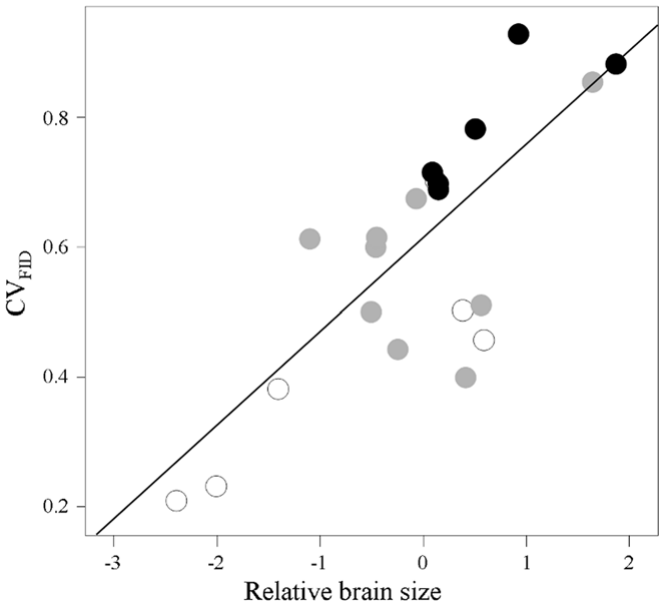
Relationship between variability in flight initiation distance (CV_FID_) measured in rural habitats and relative brain size in 21 species for which both variables were available. White dots represent species with null or occasional presence in urban sites (poor invaders), grey dots are species commonly recorded in urban sites but with higher abundances in rural habitats (successful invaders), and black dots are those showing higher abundances in urban than in rural habitats (highly successful invaders).

Furthermore, we tested the relative importance of inter-individual variability in FID and relative brain size in urban invasiveness. Generalized Linear Mixed Models showed that only CV_FID_ was significantly retained (F_1,6_ = 6.05, P = 0.049), RBS losing all explanatory power (F_1,6_ = 0.03, P = 0.86) when its role was simultaneously tested with CV_FID_. This result comes from the strong covariation between RBS and CV_FID_ (R^2^ = 0.59; F_1,8_ = 17.04, P = 0.003, Fig. 4), showing a positive relationship between inter-individual variability in FID and relative brain size. No other variable was significantly retained with CV_FID_ in these set of models, and CV_FID_ was not related to other covariates (all P>0.10).

**Figure 5:**
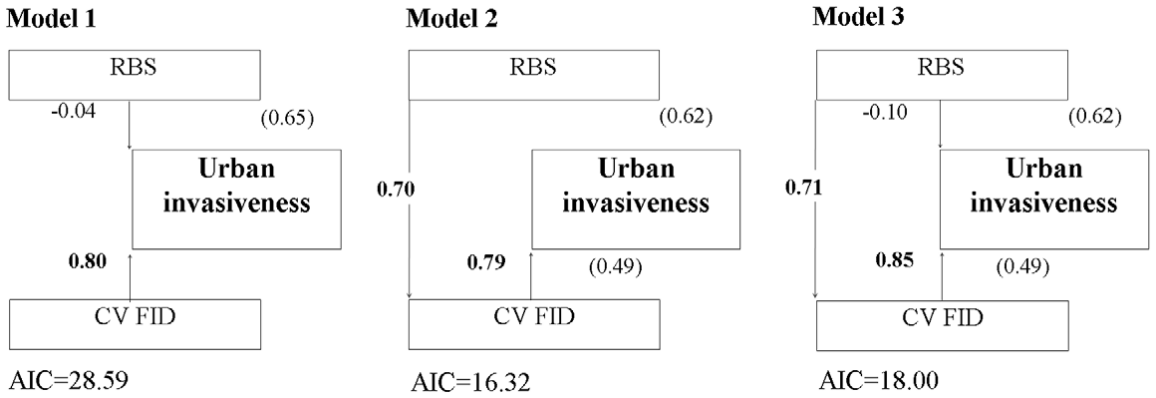
Models of hypothesized relationships between variability in flight initiation distance (CV_FID_), relative brain size (RBS) and urban invasiveness. Numbers in parentheses are the variances (*R^2^*) explained by the different models. Numbers associated with arrows are standardized factor loadings (in bold when statistically significant, all p<0.001) for the effects of variables on urban invasiveness (or CV_FID_, models 2 and 3). AIC values are provided for each model.

Finally, we constructed Confirmatory Path Analysis to ascertain the actual links between inter-individual variability in FID, RBS and urban invasiveness (see Fig. 5). In particular, we tested whether large brains and large inter-individual variability in FID may enhance urban invasiveness in an additive way (model 1), whether large brains could promote larger variability in FIDs among individuals thus increasing urban invasiveness (model 2), or whether large brains have both direct and indirect (through increased CV_FID_) positive effects on urban invasiveness (model 3). The model with lowest AIC (model 2) supports a positive effect of RBS on variability in FID that enhances urban invasiveness (Fig. 5). Although AIC differences between model 2 and 3 are <2, the lack of significance of the path from RBS to urban invasiveness in the last model makes them both biologically equivalent. These results thus suggest that large brains can promote urban invasiveness indirectly, through an increment in variability in FIDs at the species level, but not directly through enhanced cognitive abilities or other skills. Alternative models, including the rest of explanatory variables, did not include any additional statistically significant variable and showed differences in AIC>5.71 (results not shown).

## Discussion

### Fear of humans and urban invasiveness

Literature on introduced species suggests that behavioural flexibility, in the form of learning, cognition and/or rapid adjustment to new conditions, allows animals to be successful when invading novel habitats [27]–[28]. However, unlike typical biological invasions, urban areas present birds with all of the novel conditions characteristic of new environments (e.g., food resources, competitors, or breeding sites) as well as an extraordinary selective factor, i.e. humans. In this sense, our study indicates that species that are variable regarding their fear of humans and/or vehicles, which are also those with relatively larger brains, are more likely to invade urban habitats than apparently tame species. Abundant, small and generalist species also tended to show higher urban invasiveness. However, when simultaneously tested, only the effect of variability in FID remains significant, suggesting that the ability to cope with this extreme anthropogenic habitat change can be largely related to inter-individual variability in a specific behavioural trait such as fearfulness [16]. Within-species variability in fear of people thus emerges as a proximate behaviour that could explain urban invasion by a small subsample of tame individuals, while RBS could be the ultimate responsible behind the variability in this behaviour among individuals, as suggested by our confirmatory path analysis. Our results obtained from a contemporary scenario of invasion strongly support the suggestion by Møller [8] of a selection of individuals with reduced FID in urban environments.

While admitting that other unexplored behavioural traits related to brain size or FID could also contribute to explaining urban invasiveness, our results suggest that only tame individuals from variable species would cross the disturbance frontier and thus be able to live in urban environments, hence supporting the disturbance-induced habitat selection hypothesis [15]. One could ask why individuals from apparently tame species do not become urban invaders. The likely answer would be that individual fearfulness (as measured through FID) must be interpreted regarding its within-species variability. Both risk perception and the costs of fleeing from people likely vary among species, and thus just the FID of an individual tells us little about its tolerance of people if it is not compared with the variability shown by its conspecifics. In this sense, variable species seem to include some individuals which perceive human proximity as less risky than their conspecifics, being thus able to coexist with people. However, in species with low variability all individuals would be similarly affected by human disturbance, creating few opportunities for some individuals to invade urban areas.

Looking for alternative explanations to the above hypothesis, the low FIDs of urban individuals compared to their rural conspecifics could partially result from individuals habituating to human disturbance after they settled in cities, thus increasing their tameness with time. Cooke [26] found 30 years ago that birds tend to be more approachable in urban than in rural habitats, suggesting that birds in urban areas come into contact with people more often and with greater proximity, having a greater opportunity to learn within what distance a human can approach before being a danger. Since then, habituation has been often argued to explain differences in FID among areas with different human disturbance [29]. Those studies, however, were based on population means instead of on individual responses to an increased human presence. The only study so far dealing with changes in individual responses did not find evidence for a consistent short-term individual habituation to human disturbance in rural birds, using the same burrowing owl population included in this study [15]. Habituation was neither supported when relating the differences in FID between urban and rural conspecifics to time as species become urban in Old-World cities [7]. Nonetheless, further research on habituation is needed. Despite the strong within-individual consistency in FID found, some burrowing owls slightly habituated to human disturbance while others became more afraid of people [15], and we cannot discard the possibility that the relationship between individual consistency in FID and habituation could change among species.

### Brain size, individual behaviour and behavioural flexibility

The brain size-environmental change hypothesis predicts that behavioural flexibility carries fitness benefits to individuals facing novel or altered environmental conditions [17]. The principle underlying this hypothesis is the idea that enlarged brains afford advantages to individuals in dealing with environmental change when the response demands behavioural flexibility in the form of learning and innovation. Relative brain size correlates with measures of behavioural flexibility, linked to, for example, innovation capacities and the ability to deal with new environments, which could satisfactorily explain the success of several species as alien invaders [17]–[18], [28]. From these studies, one could assume that individuals from large brain species are equally flexible. However, recent research shows that species often exhibit inter-individual differences in their responses to a variety of situations such as feeding, mating, or avoiding/escaping predators. These behavioural tendencies, personality traits or behavioural syndromes (such as activity, shyness-boldness, exploration, and aggressiveness [30]–[31]) can greatly determine how these species respond to changes such as those produced by human development [16], [31]–[32].

A growing number of studies show that the majority of a population's niche width is determined by inter-individual variation [33]–[35]. Along this same line of evidence, individuals within populations show different behaviours that are heritable [36]–[37], relatively inflexible [15], [30], and linked to fitness traits, thus being favoured or disfavoured by selection depending on the particular ecological conditions experienced by the population [30], [38]. Within this context, and taking into account results presented here, behavioural flexibility should be regarded as a specific trait encompassing variability among individuals, but not necessarily within individuals. Recent studies on individual variations in FID have shown that this behaviour has a strong individual component in two bird species [15]–[16] and a reptile, *Agama planiceps* [39]. Moreover, these authors also found links between FID and other individual behaviours [16], [39], suggesting that FID can be considered as a personality trait *sensu* Réale *et al.* [30]. Therefore, although much more research is needed, we must not discard that within-individual consistency in FID could be a generalised fact in animal behaviour. No studies have been performed however to test whether FID changes across situations or is the result of early experience that then is fixed throughout life, but studies on personality envisage these possibilities for individual behaviours [31]. However, as FIDs resulted highly repeatable within individuals [15]–[16], there would be a low potential cognitive effect of larger brains on the flexibility of adult individuals (i.e., those studied here) regarding their fear of humans. Although the actual mechanism underlying the positive relationship between RBS and variability in FID found in this study remains unexplained, our results suggest an association between enlarged brains and the evolution of behavioural variability among individuals, not just the capacities of individuals to modify their behaviour in potentially adaptive ways but through increased differences in individual traits. Thus, one prominent contribution of our results is that the behaviours of individuals, but not the average behaviour at the level of species, are important during the invasion process. As previously suggested [30], further research on biological invasion processes would benefit from the perspective of variability in animal personalities.

## Materials and Methods

### Ethics Statement

Field work conducted here was not invasive and did not require the manipulation of live animals, and measuring FID from a car did not suppose an additional disturbance to that coming from daily car traffic. Brain masses were obtained from the literature and from birds found recently killed by cars in the roads. Therefore, this work did not require specific permits by the relevant Argentinean nor Spanish authorities.

### Study area

We selected the area of Bahía Blanca as a study site, on the Atlantic coast of Argentina, a relatively young city founded by European colonists in 1828. It was a small village until the middle of the twentieth century, reaching ca. 293,000 inhabitants in very recent years. The city is surrounded by natural habitats (mostly grasslands and pasturelands, with small interspersed patches of xerophytic forests and scrublands) where human presence and activities are negligible. Both pedestrian records (0–0.1 pedestrians/h) and traffic volume (0.34–2.4 cars/h) were extremely low in natural (hereafter rural) habitats when compared to typical figures for First World countries (11–325 cars/h; [1], [40]). Sampling in rural habitats was restricted to areas 20–150 km from the city to avoid potential confounding effects of urban-rural ecotones on both behaviour and relative abundance of birds (see below). Field work was conducted during wintering and summering months of 2003–2008.

### Urban invasiveness and explanatory variables

We classified species within a gradient of urban invasiveness based on their relative abundance (measured through censuses following road transects; see e.g. [41]) in urban and rural habitats. Road transects were shown to perform as well as other measurement techniques such as foot transects or point counts in estimating relative abundances of a variety of bird species in similar Argentinean open habitats [42]. In summer 2004, transects totalling 59 and 150 km were conducted in urban and rural habitats, respectively, at a constant speed (ca. 20 km/h), avoiding windy and rainy days and the hottest hours of midday. Near-road abundances (hereafter, abundances) were estimated as the number of individuals of each species recorded per km [41]–[42]. As is the case for any census methodology, differences in abundances between species may be biased by their differential detectability. In our case, smaller species could be more frequently missed when conducting road transects. This is not a problem, however, when comparing within-species abundances in rural and urban areas, since any body size bias should be common to both areas. After calculating the difference in abundance in rural minus the abundance in urban areas for each species (i.e., within-species relative abundance), urban invasiveness was scored as null or occasional (species recorded in rural habitats that were never or very rarely seen at urban sites), medium (species commonly recorded at urban sites but with higher abundances in rural habitats), and high (species showing higher abundances in urban compared to rural habitats). We chose these rough scores because urban invasion is a contemporary process in our study area: several species have become urban within the last 5–15 yr, while urban populations of others continue to increase (Authors unpubl. observations). Therefore, relative abundances may not have been established in some species and thus the use of finer scoring scales could imply their incorrect categorization. Nonetheless, the use of five scores for the categorization of urban invasiveness gave similar results to those presented in this paper. For the same reason (contemporary, dynamic process), we did not differentiate between potential urban exploiters and urban adapters (i.e., species living in the city proper and species living in the surrounding, less urbanized areas, respectively) as defined by Kark *et al.* [43]. Nonetheless, both types of species do not seem to differ in terms of behavioural flexibility as measured through relative brain size and the number of feeding innovations [43].

We recorded FIDs of different birds to car approach as a measure of the ability of individuals to cope with human disturbance. We are confident that we mostly sampled different individuals since 1) we surveyed ca. 750 km of different unpaved roads and streets across a large study area (ca. 5,000 km^2^), and 2) road surveys covered a number of territories of territorial species, such as the burrowing owl, that we identified in the course of other field-work tasks [15]. Therefore, the likelihood of resampling individuals for FID was negligible. To measure FID, we drove a small grey car at a slow, steady speed (ca. 10 km/h). Birds measured were typically perched close to (usually within 15 m) unpaved roads in rural habitats or streets at urban sites. When we selected a focal bird we did not stop for the identification of the species but approached it driving through the route at the same speed until it flew. Therefore, the approach was nearly directional (i.e., following the straight road or street in direction to the bird) and was done in the same way that usual car traffic unintentionally approaches birds. If the focal bird was close to others, we only measured FID from the focal one. Then, one of the two authors seated in the front of the car identified the species, using binoculars if needed, and measured the FID of the focal bird through the closest open window. Therefore, we cannot differentiate bird responses to the car from those to humans that were clearly visible to birds. Nonetheless, FIDs of one of the species obtained by an approaching human [15] give similar results to those when measured from a car (Authors, unpublished data). FIDs were obtained only from adult birds, discarding fledglings and juveniles (whose flight skills could be compromised by their young ages). Each FID was measured by using a LEICA laser distancemeter (measuring range: 10–800 m; accuracy: ±1 m) or through direct measurement (for distances<10 m), thus obtaining the actual Euclidean distance [9]. We recorded 1,393 FIDs from 61 species in rural habitats and 691 FIDs from 41 species in urban habitats (Figure S1), individual FIDs ranging from 0.5 to 122 m. However, to avoid potential biased results associated with small sample sizes, statistical analyses (see below) were performed following Cooke [26] and Blumstein [9] by using information from species for which we obtained FIDs from at least 10 individuals measured in rural habitats (n = 42 species, see Figure S1). As a measure of within-species variability in FID in rural habitats, we used the coefficient of variation (CV_FID_) instead of the variance because, contrary to this measure, CV was not related to body size (Regression of variance against body size: R^2^ = 0.25, *n* = 42, P = 0.0015; Regression of CV_FID_ against body size: R^2^ = 0.003, n = 42, P = 0.93). Thus, we can test the relative importance of CV_FID_ and body mass including them simultaneously in the same models.

Information on overall brain masses (in grams) was available for 27 species (44, D. Sol, unpubl. data, Authors unpubl. data). As is the case in most comparative works on brain size, the number of individuals from which brain size and body mass is available per species was generally low (see Figure S1). However, variance in both brain size and body mass is much higher among than within bird species, which is required for the feasibility of a comparative analysis [44]. Body masses were obtained from the same sources as available brain masses, completed with information from published body mass compilations [45]–[47] and own unpublished data for the rest of species. Following Sol *et al.* [17], we calculated the residuals of a log-log linear regression of brain mass against body mass. The relationship was positive and linear (linear regression, R^2^ = 0.75; F_1,25_ = 75.08, P<0.0001) and the residuals were uncorrelated to body mass (r = 0.003, P = 0.99, n = 27), hence, we used them as a measure of relative brain size [17].

The abundance of each species in rural areas was estimated as the average number of birds/km (see above). As previously mentioned, larger species could be more easily detected from a car than smaller ones. Contrary to when we previously estimated within-species relative densities in urban and rural areas, the between-species relative densities obtained in rural areas could be seriously affected by such a bias. However, abundances in our study species in rural habitats were uncorrelated to our measure of body size (i.e., body mass; r_s_ = 0.014, P = 0.93, n = 42), despite the fact that sampled species widely differed in size (body masses ranging from 13 to 890 g, see Figure S1).

Dietary and habitat generalisms were obtained as the number of the major food/habitat types recorded in the literature for each species (diet: grasses and herbs, seeds and grains, fruits and berries, pollen and nectar, vegetative material, invertebrates, vertebrates, and carrion; habitat: forest, mixed scrub, grassland, marsh and wetland, and cultivated and farm lands, [17], [48]). Environmental tolerance was calculated as the whole latitudinal distribution of each species (in degrees) including their breeding and non-breeding ranges [25].

### Statistical analyses

Generalized Linear Mixed Models (GLMMs, using the cumulative logit link function and the multinomial error distribution for categorical, ordered data; GLIMMIX procedure in SAS 9.1) were performed to investigate the relative influence of our explanatory variables on urban invasiveness (as a categorical, ordered variable with three levels; see above) while controlling for potential phylogenetic effects (Family and Order as nested random factors, following [17]). The same random factors were fitted when testing in a GLMM the relationship between CV_FID_ and brain size of the species, using a normal distribution of errors and the identity link function. As FIDs did not differ between seasons and years within species and habitats (all P>0.36), data were pooled for analyses. CV_FID_ and average FID were not significantly related (r = 0.13, P = 0.40, n = 42), so we included both variables in the same models without problems of colinearity. We compared alternative models (Fig. 5) to ascertain the links between relative brain size, CV_FID_ and urban invasiveness through Confirmatory Path Analysis, using Structural Equation Modelling (SEM) in AMOS 5. The Akaike's information criterion (AIC) was used for model selection, using differences in AIC scores (lower scores indicated greater statistical support). Models with AIC scores differing from that of the lowest score by more than two were considered to be unsupported statistically [49]. Finally, a Paired t-test was used to compare FIDs of urban and rural conspecifics for the 20 species from which we sampled at least 10 individuals in both habitats.

## Supporting Information

Figure S1 Species, degree of urban invasiveness, body mass (in g), overall brain mass (in g), mean flight initiation distances (FID, in m) for urban and rural birds, coefficient of variation (CV) of FID among rural birds, and number of FIDs measured in urban and rural areas surrounding Bahia Blanca, Argentina. For body mass and brain mass, sample size (in brackets) and source (as superscript) are shown.
(DOC)
