# Supplementary material for: Inter-Individual Variability in Fear of Humans and Relative Brain Size of the Species Are Related to Contemporary Urban Invasion in Birds
Source: PLoS One. 2011 Apr 19;6(4):e18859. doi: 10.1371/journal.pone.0018859 (PMC3079730; doi:10.1371/journal.pone.0018859)
Supplement: Figure S1 — Species, degree of urban invasiveness, body mass (in g), overall brain mass (in g), mean flight initiation distances (FID, in m) for urban and rural birds, coefficient of variation (CV) of FID among rural birds, and number of FIDs measured in urban and rural areas surrounding Bahia Blanca, Argentina. For body mass and brain mass, sample size (in brackets) and source (as superscript) are shown. (DOC) [file pone.0018859.s001.doc]

| **Species** | **Urban invasiveness** | **Body**  **mass** | **Brain**  **mass** | **Urban FID** | | **Rural FID** | | |
| --- | --- | --- | --- | --- | --- | --- | --- | --- |
|  |  |  |  | **Mean** | **N** | **Mean** | **CV** | **N** |
| Agelaius thilius* | 0 | 30 (1)^2^ |  |  | 0 | 5.00 |  | 1 |
| Ammodramus humeralis | 0 | 17 (12)^2^ |  |  | 0 | 10.41 | 0.35 | 29 |
| Anthus furcatus* | 0 | 20 (29)^2^ |  |  | 0 | 6.50 |  | 2 |
| Anumbius annumbi | 0 | 41.5 (2)^2^ | 1.22 (3)^5^ | 7.00 | 1 | 10.13 | 0.71 | 27 |
| Aratinga acuticaudata* | 1 | 173 (8)^6^ | 5.44 (3)^5^ |  | 0 | 24.00 |  | 8 |
| Asio flammeus* | 0 | 347 (47)^2^ | 5.45 (4)^1^ |  | 0 | 25.00 |  | 7 |
| Athene cunicularia | 2 | 155 (46)^2^ | 3.92 (10)^5^ | 5.40 | 41 | 15.69 | 0.94 | 78 |
| Bubulcus ibis | 1 | 338 (9)^2^ | 3.95 (10)^5^ | 10.50 | 2 | 22.00 | 0.68 | 14 |
| Buteo magnirostris* | 1 | 269 (16)^2^ | 4.72 (11)^5^ | 10.08 | 6 | 19.43 |  | 7 |
| Buteo polyosoma | 0 | 950 (?)^3^ | 9.10 (3)^5^ |  | 0 | 39.17 | 0.51 | 15 |
| Buteo swainsoni* | 0 | 988 (12)^2^ |  |  | 0 | 32.50 |  | 4 |
| Carduelis magellanica | 2 | 11 (2)^2^ |  | 3.75 | 14 | 16.09 | 0.59 | 11 |
| Cathartes aura* | 0 | 1467 (20)^2^ |  |  | 0 | 79.00 |  | 1 |
| Colaptes campestris | 1 | 165 (5)^2^ |  | 7.16 | 16 | 17.92 | 0.56 | 46 |
| Colaptes melanochloros | 1 | 121 (18)^2^ |  | 7.74 | 17 | 13.90 | 0.63 | 15 |
| Columba maculosa | 2 | 347 (4)^2^ |  | 3.60 | 40 | 20.47 | 0.81 | 19 |
| Columba picazuro | 2 | 402 (1)^2^ |  | 6.53 | 39 | 27.47 | 0.54 | 38 |
| Columbina picui | 2 | 46 (54)^6^ |  | 3.64 | 56 | 14.34 | 0.66 | 44 |
| Cyanoliseus patagonus | 2 | 273 (4)^2^ | 8.53 (3)^5^ | 7.42 | 12 | 31.63 | 0.89 | 38 |
| Drymornis bridgesii* | 0 | 95 (2)^2^ |  | 12.50 | 2 | 23.00 |  | 1 |
| Elanus leucurus | 1 | 300 (?)^3^ |  | 25.00 | 2 | 36.60 | 0.39 | 10 |
| Embernagra platensis | 0 | 46 (1)^4^ |  |  | 0 | 11.79 | 0.25 | 19 |
| Eudromia elegans | 0 | 660 (1)^2^ | 2.40 (10)^5^ |  | 0 | 2.45 | 0.24 | 21 |
| Falco femoralis | 0 | 334 (13)^2^ |  | 17.00 | 1 | 32.76 | 0.20 | 17 |
| Falco sparverius | 1 | 115 (180)^2^ | 2.58 (23)^5^ | 13.25 | 12 | 27.84 | 0.41 | 74 |
| Furnarius rufus | 2 | 51 (37)^6^ | 1.40 (3)^1,6^ | 3.13 | 57 | 12.45 | 0.71 | 30 |
| Geranoaetus melanoleucus* | 0 | 2252 (2)^2^ |  |  | 0 | 23.67 |  | 3 |
| Griseotyrannus aurantioatrocristatus | 0 | 27.2 (3)^2^ |  |  | 0 | 16.10 | 0.20 | 10 |
| Guira guira | 1 | 146 (5)^5^ | 1.99 (5)^5^ | 4.35 | 10 | 14.25 | 0.61 | 32 |
| Hymenops perspicillatus* | 0 | 24 (2)^2^ | 0.79 (4)^5^ |  | 0 | 25.00 |  | 5 |
| Lessonia rufa | 0 | 14 (4)^2^ |  |  | 0 | 4.85 | 0.21 | 10 |
| Machetornis rixosus* | 2 | 33 (6)^2^ |  | 4.00 | 1 |  |  | 0 |
| Milvago chimango | 2 | 296 (29)^2^ | 4.76 (3)^5^ | 3.31 | 78 | 20.78 | 0.79 | 107 |
| Mimus saturninus | 2 | 73 (1)^2^ |  | 4.03 | 44 | 15.54 | 0.77 | 38 |
| Mimus triurus | 0 | 52 (2)^2^ |  |  | 0 | 27.80 | 0.21 | 15 |
| Molothrus badius | 1 | 43 (1)^4^ | 1.53 (5)^5^ | 6.44 | 8 | 8.09 | 0.52 | 16 |
| Molothrus bonariensis | 2 | 52 (53)^6^ |  | 4.80 | 38 | 11.34 | 0.76 | 28 |
| Myiopsitta monachus | 1 | 101 (9)^2^ | 4.23 (5)^5^ | 11.50 | 6 | 20.61 | 0.86 | 33 |
| Neoxolmis rufiventris* | 0 | 77(1)^2^ |  |  | 0 | 21.00 |  | 5 |
| Nothura darwini | 0 | 250 (1)^6^ |  |  | 0 | 1.70 | 0.15 | 10 |
| Nothura maculosa | 0 | 300 (1)^2^ | 1.25 (2)^6^ |  | 0 | 2.62 | 0.22 | 26 |
| Phytotoma rutila | 0 | 32 (1)^2^ |  |  | 0 | 11.18 | 0.25 | 14 |
| Pitangus sulphuratus* | 2 | 61 (15)^2^ | 1.32 (3)^1^ | 9.47 | 19 | 20.33 |  | 9 |
| Polyborus plancus | 0 | 893 (24)^2^ | 9.59 (6)^5^ | 20.00 | 2 | 41.60 | 0.47 | 48 |
| Progne elegans* | 1 | 49 (?)^2^ |  | 3.50 | 2 | 10.00 |  | 2 |
| Progne tapera* | 2 | 36 (1)^2^ |  | 3.23 | 11 | 7.67 |  | 3 |
| Pseudoseisura lophotes* | 0 | 86 (6)^2^ |  | 6.00 | 2 |  |  | 0 |
| Pyrocephalus rubinus | 1 | 14 (11)^2^ | 0.48 (7)^5^ | 8.96 | 12 | 22.22 | 0.51 | 31 |
| Serpophaga subcristata* | 0 | 7 (5)^2^ |  | 2.00 | 1 |  |  | 0 |
| Sicalis flaveola | 2 | 17 (46)^6^ | 0.73 (6)^5^ | 2.33 | 3 | 9.80 | 0.70 | 10 |
| Sicalis luteola* | 1 | 10 (32)^6^ | 0.50 (6)^5^ | 4.58 | 6 | 14.00 |  | 7 |
| Sturnella defilippi | 0 | 100 (1)^6^ |  |  | 0 | 15.41 | 0.38 | 22 |
| Sturnella loyca | 0 | 113 (?)^2^ | 1.10 (1)^6^ |  | 0 | 15.67 | 0.39 | 36 |
| Suiriri suiriri | 1 | 15 (6)^2^ |  |  | 0 | 14.27 | 0.42 | 11 |
| Syrigma sibilatrix* | 0 | 370 (1)^2^ |  |  | 0 | 43.50 |  | 2 |
| Tachycineta meyenii* | 1 | 18 (?)^2^ |  | 8.00 | 5 |  |  | 0 |
| Thraupis bonaerensis* | 0 | 36 (41)^2^ |  |  | 0 | 7.00 |  | 1 |
| Troglodytes aedon* | 1 | 11 (346)^2^ |  |  | 0 | 3.50 |  | 1 |
| Turdus falcklandii* | 2 | 94 (?)^2^ |  | 4.10 | 10 | 15.00 |  | 1 |
| Tyrannus melancholicus | 1 | 38 (35)^2^ |  | 6.39 | 9 | 12.09 | 0.51 | 23 |
| Tyrannus savana | 1 | 29 (13)^2^ | 0.76 (8)^5^ | 4.69 | 18 | 11.18 | 0.62 | 65 |
| Vanellus chilensis | 1 | 327 (3)^2^ | 2.40 (3)^5^ | 5.78 | 18 | 27.03 | 0.62 | 63 |
| Xolmis irupero | 1 | 30 (3)^2^ | 0.85 (3)^5^ | 9.31 | 13 | 23.06 | 0.45 | 17 |
| Zenaida auriculata | 2 | 114 (16)^2^ |  | 2.31 | 40 | 19.76 | 0.72 | 71 |
| Zonotrichia capensis | 2 | 21 (26)^2^ | 0.80 (2)^1^ | 4.09 | 16 | 10.58 | 0.72 | 32 |

*Species not included in analysis of urban invasiveness because of low sample size (< 10 individuals measured in rural areas). Sources of body and brain masses: ^1^Garamszegi [61], ^2^Dunning [62], ^3^del Hoyo et al [63], ^4^Salvador [64], ^5^Sol unpublished data, ^6^Authors unpublished data. (?) Unspecified sample size.
